# Production of selenium nanoparticles in *Pseudomonas putida* KT2440

**DOI:** 10.1038/srep37155

**Published:** 2016-11-15

**Authors:** Roberto Avendaño, Nefertiti Chaves, Paola Fuentes, Ethel Sánchez, Jose I. Jiménez, Max Chavarría

**Affiliations:** 1 Centro Nacional de Innovaciones Biotecnológicas (CENIBiot), CeNAT-CONARE, 1174-1200 San José, Costa Rica; 2 Escuela de Química, Universidad de Costa Rica, 11501-2060 San José, Costa Rica; 3 Centro de Electroquímica y Energía Química (CELEQ), Universidad de Costa Rica, 11501-2060 San José, Costa Rica; 4 Centro de Investigaciones en Estructuras Microscópicas (CIEMIC), Universidad de Costa Rica, 11501-2060 San José, Costa Rica; 5 Faculty of Health and Medical Sciences, University of Surrey, GU2 7XH Guildford, UK; 6 Centro de Investigaciones en Productos Naturales (CIPRONA), Universidad de Costa Rica, 11501-2060 San José, Costa Rica

## Abstract

Selenium (Se) is an essential element for the cell that has multiple applications in medicine and technology; microorganisms play an important role in Se transformations in the environment. Here we report the previously unidentified ability of the soil bacterium *Pseudomonas putida* KT2440 to synthesize nanoparticles of elemental selenium (nano-Se) from selenite. Our results show that *P. putida* is able to reduce selenite aerobically, but not selenate, to nano-Se. Kinetic analysis indicates that, in LB medium supplemented with selenite (1 mM), reduction to nano-Se occurs at a rate of 0.444 mmol L^−1^ h^−1^ beginning in the middle-exponential phase and with a final conversion yield of 89%. Measurements with a transmission electron microscope (TEM) show that nano-Se particles synthesized by *P. putida* have a size range of 100 to 500 nm and that they are located in the surrounding medium or bound to the cell membrane. Experiments involving dynamic light scattering (DLS) show that, in aqueous solution, recovered nano-Se particles have a size range of 70 to 360 nm. The rapid kinetics of conversion, easy retrieval of nano-Se and the metabolic versatility of *P. putida* offer the opportunity to use this model organism as a microbial factory for production of selenium nanoparticles.

Selenium (Se) is a metalloid element widely used in industry: applications include its utilization in electronics, production of glass and photocopying^1^,^2^,^3^,^4^. The oxidized forms of the element, such as in sodium selenite, are also used as food supplements^5^. In addition to its industrial applications, Se has several properties of medical relevance due to its antimicrobial^6^,^7^,^8^ and antioxidant activities^9^,^10^. Selenium is found primarily in four inorganic chemical forms in natural environments: selenate (SeO_4_^−2^), selenite (SeO_3_^−2^), elemental selenium (Se^0^) and selenide (Se^−2^). Se also appears in seleno-organic compounds at low levels^11^. The presence of selenium in the environment occurs as a result of natural processes and human activities^3^. Naturally occurring selenium is present in seleniferous soils and copper ores at generally minute concentrations^11^,^12^, but greater concentrations occur in other environments as a result of the numerous industrial applications^4^,^13^ mentioned above. Elemental selenium is insoluble in water, and exhibits no or little toxicity in terrestrial and aquatic environments. In contrast, selenium oxyanions (i.e. selenate, selenite, recognized as selenium soluble forms) might cause harmful effects in the environment because of their substantial solubility, mobility and toxicity^14^.

The transformation in the environment of the various forms of selenium is well documented, and so is the important role that microorganisms play during the geological cycle of this element^3^,^15^. In this context, the microbial reduction of selenate or selenite to selenium is critical to decrease the bioavailability of this element, which becomes insoluble in water when reduced to Se^0 ^16. The capability of reducing selenate and selenite in the environment seems to be widespread in the microbial world, as evidenced by the diversity of species (both anaerobic and aerobic) that display this reducing ability. These species include, among others, *Pseudomonas stutzeri, Pseudomonas fluorescens*^11^,^13^, *Pseudomonas aeruginosa*^17^, *Duganella sp*., *Agrobacterium sp*.^18^, *Enterobacter cloacae*^19^, *Bacillus selenitireducens*^20^, *Azospirillum brasilense*^21^, *Thauera selenatis*^22^, *Azoarcus sp*.^23^ and *Pantoea agglomerans*^9^.

To date, the crucial components of the global process of biotransformation have not been completely elucidated. It is thought that the reduction of selenium oxyanions involves two steps: first selenate is reduced to selenite and then selenite is reduced to elemental selenium. Two mechanisms have been reported to account for the reduction of selenate to selenite, SeO_4_^−2^ → SeO_3_^−2^. One mechanism involves the participation of nitrate or nitrite reductases. Sabaty *et al*.^24^ reported that a periplasmic nitrate reductase is responsible for the reduction of selenate in *Ralstonia eutropha, Paracoccus denitrificans* and *Paracoccus pantotrophus*; these authors also proposed that this feature is common to all denitrifying bacteria. The second mechanism requires the participation of specific selenate reductases^25^. A paradigmatic example of this is selenate reduction in *Thauera selenatis*. This Gram-negative beta-proteobacterium, which was isolated from selenium-contaminated drainage water in the San Joaquin Valley of California^26^, is a facultative anaerobe capable of using selenate as a terminal electron acceptor because of the presence of a periplasmic selenate reductase (SerABC)^27^.

Selenite is also transformed into selenium, SeO_3_^−2^ → Se^0^, by the following mechanism^28^: (i) selenite becomes chelated first by thiol-containing molecules such as glutathione, leading to the production of selenodiglutathione. (ii) this compound is the substrate of the enzyme glutathione reductase that produces unstable intermediates eventually converted to elemental selenium. This process has an impact on the physiology of the cell and during selenite reduction a large amount of peroxide is produced, thereby inducing the expression of genes related to oxidative stress resistance (superoxide dismutase, catalase, peroxidase, etc). Altogether, these findings indicated that the reduction of selenite to selenium depends strongly on the ability of a microorganism to maintain a reducing environment. The fact that various bacterial species such as *Escherichia coli* and *Rhodobacter sphaeroides* overexpress enzymes involved in the resistance to oxidative stress in the presence of selenite^29^,^30^ is hence not surprising. The mechanism of conversion of selenate or selenite into Se° seems to vary between bacterial species; a significant number of small molecules and enzymatic activities are likely to be involved *in vivo* in complex communities. Unraveling additional ways for the reduction of selenate and selenite is relevant to the understanding of the biochemistry of Se in the environment, the explotation of microorganisms as biofactories of selenium compounds and the bioremediation of niches contaminated with selenate or selenite.

The size of the Se^0^ particles is an important factor that determines their potential chemical or biological activity. Nano-Se particles smaller than 100 nm have greater antioxidant activity than larger particles^9^. Nano-Se particles of size 5–200 nm can directly scavenge free radicals *in vitro* depending on their size^31^. Microorganisms can produce Se^0^ nanoparticles ranging from 30 nm^1^,^9^ to 500 nm^1^,^32^ depending on the reducing species and conditions. The microbial production of nanoparticles can, therefore, be seen as an opportunity not only for bioremediation but also in nanobiotechnology due to the multiple applications of this element^33^,^34^.

In this work, we studied the biosynthesis of nano-Se by soil bacterium *Pseudomonas putida* KT2440^35^, which is widely used in environmental applications. *P. putida* has many advantages for its use in biotechnology: it is a safe non-pathogenic bacterium^36^, it is easily cultured and manipulated in the laboratory, and it displays great metabolic versatility. As it has recently been reported, the central metabolism is equipped with the enzymes necessary to produce a high yield of reducing power (i.e. NADPH equivalents)^37^,^38^,^39^. This capability is very relevant for reducing selenite, making *P. putida* an attractive and versatile microbial species suitable for applications of this biocatalytic process.

## Results and Discussion

### *P. putida* reduces selenite but not selenate

**Figure 1:**
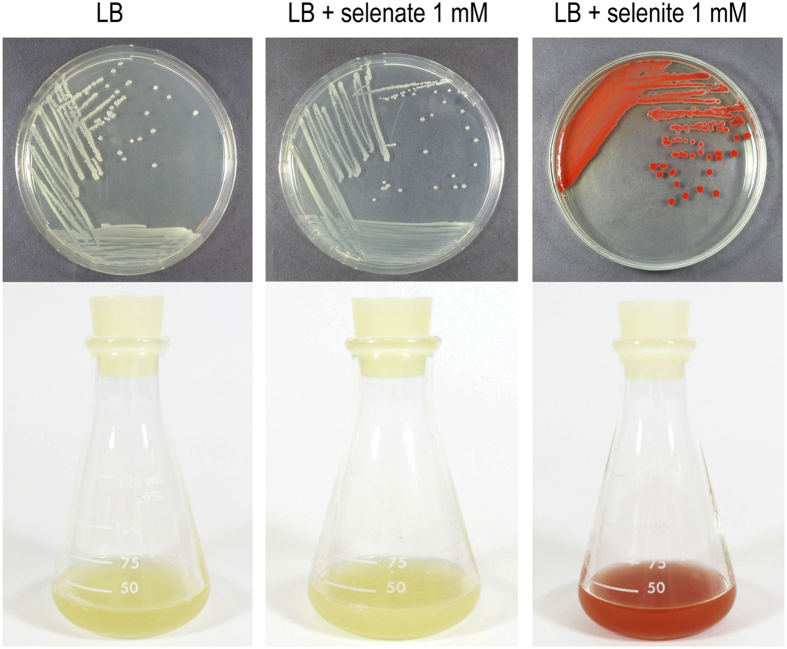
Growth of *P. putida* KT2440 in LB broth in absence and presence of selenate or selenite (1 mM). Liquid and solid cultures showed that reduction to red elemental selenium occurred only in the presence of selenite. Photographs were taken after culturing for 24 h.

**Figure 2:**
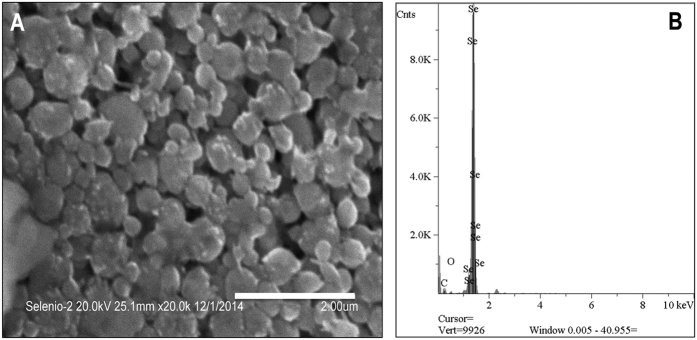
SEM-EDS analysis of Nano-Se. **(A)** SEM micrographs of dried selenium nanoparticles on a 2-μm scale. Micrographs indicate that nanoparticles agglomerated to form vesicles of an average size of 550 nm. (**B**) EDS analysis of nano-Se shows the recovered material was constituted by selenium.

To test whether *P. putida* is capable of reducing aerobically selenate and selenite to elemental selenium, we performed growth experiments in rich medium (LB; Fig. 1) and minimal medium (M9, citrate 0.2% w/v; data not shown) in the presence and absence of each oxyanion SeO_3_^−2^ or SeO_4_^−2^ (1 mM). Cultures exhibiting a characteristic red precipitate formed only in the presence of selenite (Fig. 1). This precipitate was purified as described in the experimental section and analyzed with SEM-EDS (Fig. 2). Micrographs of the precipitate (Fig. 2A) show the presence of particles with a significant heterogeneity in size. Further inspection of these particles confirmed that they were composed of elemental Se and products containing carbon and oxygen (Fig. 2B).

As shown in the growth experiments in Fig. 1, *P. putida* is not capable of reducing selenate ions to elemental selenium neither in LB nor in M9 minimal medium (not shown). These results indicated that *P. putida* KT2440 lacks the enzymatic complement needed for selenate respiration and to perform the first step of reduction, SeO_4_^−2^ → SeO_3_^−2^. The apparent absence of selenate reductase activity in *P. putida* is expected as this bacterium is considered a strict aerobe and, as such, there is no known substrate other than oxygen that might serve as a final electron acceptor. The ability to reduce the selenate ion has been associated mainly with anaerobic bacteria that can use SeO_4_^−2^ as a terminal electron acceptor^25^,^40^. Only a few cases of bacteria capable of aerobically reducing both selenate and selenite exemplified by *P. stutzeri* have been reported^13^. This bacterium was isolated from a contaminated site with high concentrations of selenium oxyanions and reflects the adaptation capabilities of the *Pseudomonas* genus.

As expected, because of the differences between cell growth rates in rich (LB) and minimal media (M9 citrate), red elemental selenium appeared in LB after culturing for 15 h, whereas in M9 citrate it occurred after nearly 72 h (data not shown). For this reason, and also due to the inability of *P. putida* to reduce selenate, we focused the following experiments exclusively on the reduction of selenite in LB.

### Toxicity of selenite and kinetics of reduction

**Figure 3:**
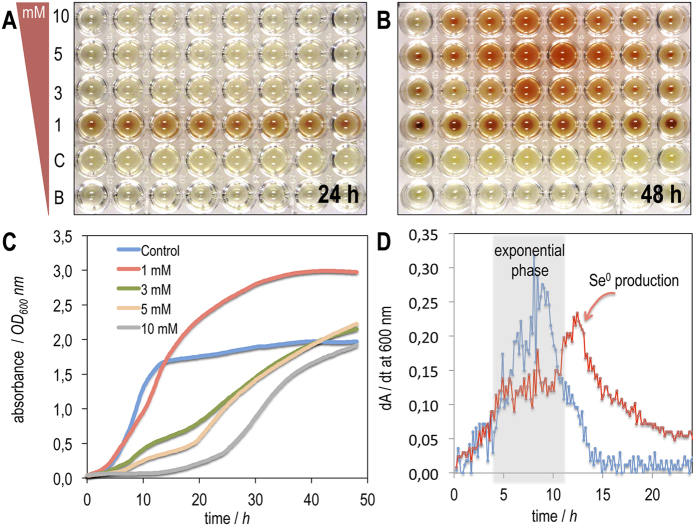
Growth and selenite reduction of *P. putida* KT2440 in LB broth with sodium selenite at different concentrations. Growth and selenite reduction was monitored over time in microtitre plates using initial concentrations of selenite of 0–10 mM after **(A)** 24 h and **(B)** 48 h. The photographs show that after culturing for 24 h the reduction is visible only when using a concentration of 1 mM selenite, but after 48 h the red elemental selenium is perceptible at all tested concentrations. **(C)** Growth curve of *P. putida* KT2440 in LB broth supplemented with selenite (0–10 mM). The curves show a significant inhibition of growth at concentrations above 3 mM. The increased absorbance in the presence of selenite (1 mM) with respect to the results of a control are due to the scattering and absorption of radiation of selenium particles. (**D)** Growth fingerprinting of *P. putida* KT2440 in the absence and presence of selenite (1 mM). The presence of a second signal in the growth fingerprinting in the presence of selenite indicates the reduction begins between 10–15 h, i.e. the middle of the exponential phase.

To study the toxicity of selenite in *P. putida* KT2440 we performed growth experiments in LB medium at varied selenite concentrations (see Fig. 3A–C). As shown, selenite (1 mM) seems optimal for our experiments because (i) greater concentrations of the oxyanion inhibit bacterial growth (Fig. 3C) and (ii) the accumulation of elemental selenium was evident in less than 24 h, whereas greater concentrations of selenite required longer incubations (24–48 h) in order to produce visible pellets (see Fig. 3A–C). Selenate had no inhibitory effect on bacterial growth when used in identical experimental conditions (data not shown).

A detailed examination of the growth curve in Fig. 3C shows how, in the presence of selenite (1 mM), the absorbance attained by the culture exceeded the maximum value obtained by the strain in the absence of selenite. Since elemental selenium scatters and absorbs radiation at 600 nm (at which the absorbance is measured), the increased apparent absorbance is due not only to the increased biomass but also to the elemental selenium accumulated. We took advantage of this property to monitor the growth phase in which reduction begins. For this purpose, we generated the growth fingerprints^41^,^42^ of the cultures using the curves of Fig. 3C. Such growth fingerprints allow the sensitive detection of changes during cell growth by generating the first-derivative (dA/dt vs t) of the growth curve. Growth fingerprints of *P. putida* KT2440 in the absence (control) and presence of selenite (1 mM) are shown in Fig. 3D. The results show that when selenite is absent, a single feature is observed between 6–10 h that corresponds to the slope generated in the exponential growth phase, whereas in the presence of selenite (1 mM) two signals were observed: a gradual first signal at 4–8 h and a sharp second feature at 10–15 h. These results indicate that the two signals corresponded, respectively, to the exponential growth phase of the cells, and to the increased absorbance generated by the production of selenium (centred at 12 h). This behaviour is consistent with the reduction of selenium beginning in the mid-exponential phase of growth.

**Figure 4:**
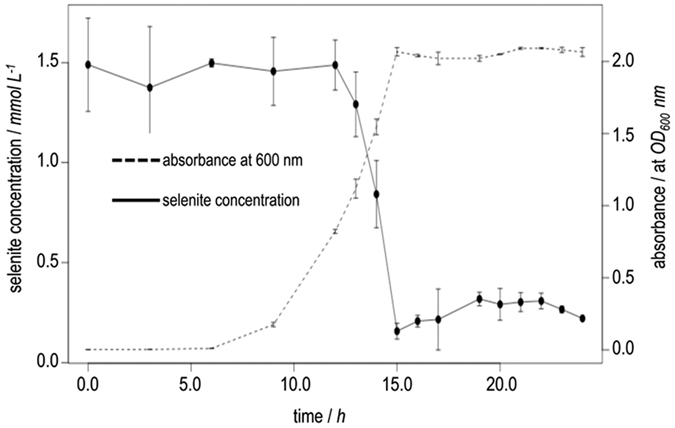
Kinetics of bacterial growth and selenite depletion of *P. putida* KT2440. We determined selenite using ICP-OES as described in the methods section. The results are consistent with the reduction beginning in the mid-exponential phase as indicated by growth fingerprinting. As seen, selenite (initial conc. 1.5 mM) is reduced in just 3 h (12–15 h) with a total conversion of 89%. Plots show the mean values and standard deviations of two independent experiments.

This observation was further validated by monitoring the variation of the selenite concentration in the culture medium (Fig. 4). Aliquots of the culture were withdrawn at periodic intervals, centrifuged and filtered with mixed cellulose ester membrane filters (pore size 0.20 μm). This purification step removed cells and insoluble aggregates of Se^0^ so that only selenite remained in solution. The supernatants were analyzed using inductively coupled plasma optical emission spectra (ICP-OES). The reduction kinetics determined by ICP-OES (Fig. 4) agreed with the estimation made in the fingerprints of the growth experiment (Fig. 3D), confirming that the reduction began in the mid-exponential phase. The data showed also an abrupt decrease in selenite concentration once the genes involved in the reduction were activated. The process occurred rapidly with selenite shifting from ~1.49 mM to ~0.16 mM (i.e. approx 89% of conversion) in just 3 h (12–15 h) at a rate of 0.444 mmol selenite L^−1^ h^−1^. The rate of conversion of *P. putida* was greater than the rates obtained using other microorganisms: *Bacillus cereus* 0.125 mmol selenite L^−1^ h^−1 ^43, *Bacillus mycoides* 0.08 mmol selenite L^−1^ h^−1 ^32, *Comamonas testosteroni* 0.014 mmol selenite L^−1^ h^−1 ^44 and *P. stutzeri* 0.175 mmol selenite L^−1^ h^−1 ^13. These rates were calculated from data available in the literature making sure (i) that the corresponding experiment was performed under similar conditions, i.e. monitoring simultaneously the bacterial growth and the concentration of selenite, and (ii) the initial concentration of selenite was in a range of 0.5 to 2 mM. In each case the slope was calculated as the change in concentration of selenite per unit of time in a linear interval ranging from the beginning of reduction until the end, determined as the first point of the plateau at which the selenite concentration remained constant (not neccesarily equal to zero). Despite the limited amount of data available, these results allow us to argue that *P. putida* is among the known bacteria that have a significant capacity to reduce selenite to nano-Se. Although these experiments were performed with 1 mM selenite, *P. putida* KT2440 is capable of reducing concentrations of selenite 10 times higher in 48 hours, enduring similar levels to *T. selenatis*^22^, which is considered a model organism for selenium metabolism and has been proposed as a suitable system for bioremediation of selenium oxyanions^45^.

### Characterization of selenium nanoparticles

**Figure 5:**
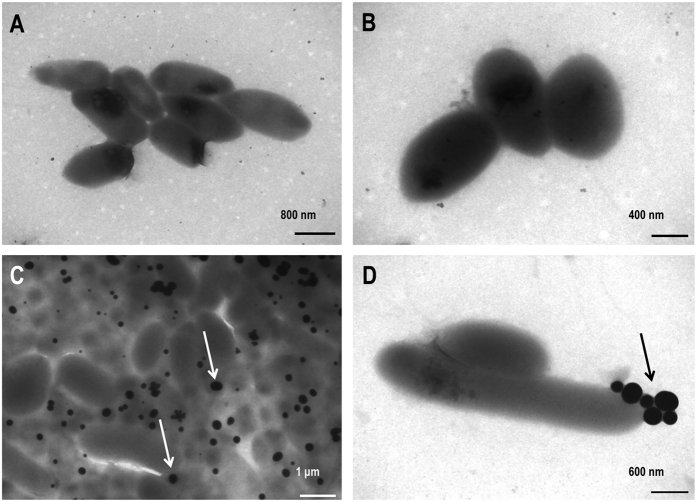
TEM images of *P. putida* KT2440 in (**A,B**) the absence and (**C,D**) presence of selenite. The micrographs show abundant electro-dense spheres (some marked with arrows) corresponding to accumulated selenium nanoparticles when the cells were cultured in the presence of selenite.

We next characterized the selenium particles produced by *P. putida* KT2440. For this purpose, we collected culture samples (in absence and presence of selenite at 1 mM) after culturing the cells for 24 h and once the accumulation of red Se^0^ was perceptible. Samples were processed as described in the experimental section and analyzed with a transmission electron microscope (TEM); results are summarised in Fig. 5. The micrographs show an accumulation of electron-dense particles attached to the outer side of the external cell membrane or in the immediate vicinity of the cells cultured in the presence of selenite (Fig. 5C,D). These particles were not observed in the cells grown on LB without the oxyanion (Fig. 5A,B). Despite the extensive accumulation of Se^0^ with a tendency to form aggregates of individual particles (see Fig. 5D), no evidence of cell lysis or distortion of the outer membrane was observed. The particles appeared to have a spherical shape and a diameter of approximately 100–500 nm with an average of 266 nm, similar to those found in *Duganella sp*., *Agrobacterium sp*.^18^, *E. cloaocae*^19^, *B. selenitireducens*^20^, and *T. selenatis*^22^.

**Figure 6:**
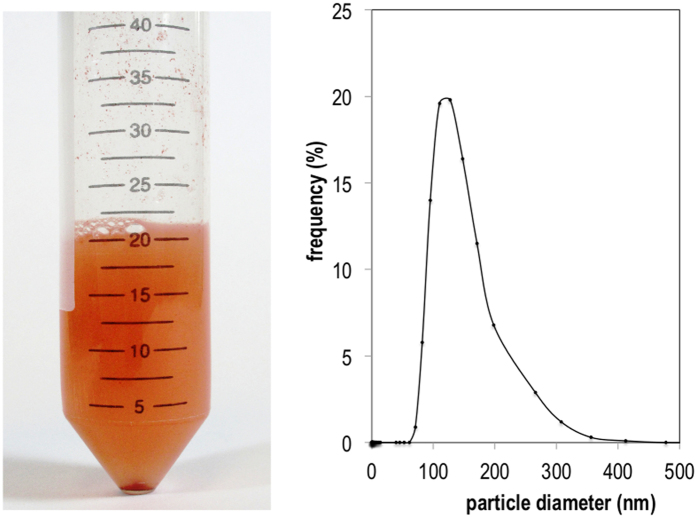
Dynamic light scattering (DLS) of the aqueous suspension of purified nano-Se. An aqueous suspension of recovered nano-Se (left panel) was prepared and analyzed by DLS as described in the methods section. A plot of the frequency distribution of diameters in a suspension of particles (right panel) shows a range of particle sizes from 70 to 360 nm.

We determined the size distribution and purity of selenium nanoparticles after the recovery process using, respectively, dynamic light-scattering (DLS) and inductively coupled plasma optical emission spectrometry (ICP-OES). DLS results are shown in Fig. 6. The particles had sizes ranging from 70 to 360 nm with a mass percent of selenium in the recovered solid of 15 ± 3%. The discrepancy between the mass percentage of selenium obtained by ICP-OES and the results obtained by SEM-EDS (Fig. 2B) can be associated to the semiquantitative nature of the latter, which only analyses specific parts of the sample. Higher yields of recovery might be obtained by developing a purification method that could remove organic matter from the sample. The size distribution obtained by DLS is close to the range of particle diameter determined from TEM micrographs of the bacterial culture (100–500 nm). The wide range of particle sizes does not allow us to conclude whether significant agglomeration occurs during the recovery step. Previous studies^1^ have reported that agglomeration of Nano-Se particles takes place spontaneously over time. This process may be prevented in the presence of ligands such as albumin and poly-vinylpyrrolidone (PVP)^1^. Given the importance of particle size in determining the chemical and biological properties, it would be of interest to explore the possibility of using ligands to control the size of Se° nanoparticles produced by *P. putida*.

In conclusion, the soil bacterium *P. putida* KT2440 is able to produce nano-Se from selenite in an environmentally compatible process, avoiding the traditional and costly reduction that employs chemical products and high temperature. Although the nature of the enzymatic activities responsible for the reduction is unknown, *P. putida* KT2440 clearly works as an effective system to produce nano-Se that has advantages over other microorganisms previously reported. *P. putida* KT2440 is a well-known microorganism that grows in inexpensive culture media and for which there are molecular tools for genetic engineering. Perhaps most importantly, the metabolism of this species is adapted to produce a high reducing power^37^,^38^,^39^. This is the consequence of having a specialized metabolism coupling enzymes from the Entner-Doudoroff path, the Embden–Meyerhof–Parnas path (i.e. glycolysis) and the Pentose Phosphate path^38^,^39^. These metabolic steps allow the generation of NADH/NADPH at large concentrations and give *P. putida* KT2440 a greater resistance to oxidative stress compared to other bacteria, such as *E. coli* or *Agrobacterium tumefaciens*^37^. This property makes *P. putida* more suitable to catalyse reduction processes such as the synthesis of selenium from selenite.

## Methods

### Bacterial strains, culture media and growth conditions

*P. putida* KT2440 was grown aerobically at 30 °C with orbital shaking at 170 rpm either in LB broth^46^ or in M9 medium^47^ containing citrate (0.2% w/v) as the sole source of carbon and energy. Cultures in plates were prepared using the same media supplemented with Bacto Agar (1.5% w/v, Pronadisa Madrid, Spain). When required, sodium selenite or sodium selenate (Sigma-Aldrich WGK, Germany) was added at the required concentration (1–10 mM).

### Recovery of selenium nanoparticles from the culture broth *P. putida*

KT2440 was cultured in LB medium supplied with selenite (1 mM) at 30 °C and 170 rpm. After 24 h, the culture (50 mL) was centrifuged (10 min, 4,000 × *g*, 4 °C) in a conical tube to form a pellet comprising both the cells and the selenium particles. Upon removal of the supernatant, the pellet was resuspended in water and vortexed for 5–10 s. In this step the cells were primarily transferred to the supernatant while the remaining pellet was composed mainly of particles of selenium. This washing step was repeated three times, after which the remaining pellet was dried at 25 °C.

### Scanning Electron Microscope and Electron Dispersive Spectrometer (SEM-EDS)

The chemical elements that constitute the purified nanoparticles were determined with SEM-EDS. For that purpose, recovered pellets of selenium particles were analyzed with a scanning electron microscope (Hitachi S-570, Tokyo, Japan) with energy-dispersive X-ray spectra (SEM-EDS).

### Selenite sensitivity tests

To establish suitable conditions to analyze the production of selenium nanoparticles we grew pre-inocula of *P. putida* KT2440 overnight in LB as described above. These cultures were diluted to an initial absorbance of approximately 0.05 at 600 nm (OD_600_) in fresh medium containing sodium selenite (0, 1, 3, 5 or 10 mM). Bacterial growth was estimated by monitoring OD_600_ (Synergy H1 Hybrid Multi-Mode Reader, Biotek, Winooski VT, USA) of cultures in plates (96 wells; Nunclon™Δ Surface; Nunc A/S, Roskilde, Denmark). Plates were incubated at 30 °C for 48 h with continuous orbital shaking and the absorbance was measured every 10 min.

### Kinetics of selenite reduction

Cells were cultured in baffled conical flasks (3 L) filled with LB (500 mL) and supplemented with sodium selenite (1 mM) at 30 °C and 170 rpm. Cell suspension aliquots (15 mL) were withdrawn at intervals during the bacterial growth for measurement of their OD_600_. Cells in these aliquots were removed by centrifugation (10 min, 4,000 × *g*, 4 °C); the resulting supernatants were transferred to a new tube (15 mL) and stored at −25 °C until further analysis. Supernatants were filtered using mixed cellulose ester filters (ADVANTEC^®^, 0.20 μm), and the concentration of selenite was determined using an inductively coupled plasma optical emission spectrometer (ICP-OES, Perkin-Elmer, model 400, Norwalk, CT, USA). The equipment was operated in an axial configuration at wavelength 196.026 nm for selenium. The analyte solutions were prepared from standard solutions (1000 mg/L, Tritrisol-Merck, Darmstadt, Germany). Appropriate dilutions were made to prepare the standards, which were stored in polyethylene flasks under refrigeration.

### Transmission electronic microscope (TEM)

*P. putida* KT2440 strain was streaked in LB agar plates supplemented with selenite (1 mM). After growth for 24 h, a swab was taken and fixed for 12 h at 4 °C in Karnovsky’s fixative^48^. An aliquot (100 μL) of this solution was placed on a copper grid (formvar-coated, 400 mesh) and dried on a filter paper at 25 °C. Grids were examined wtih a transmission electron microscope (Hitachi H-7100, Tokyo, Japan) at an accelerating voltage of 100 kV.

### Dynamic light-scattering (DLS)

The sizes, reported as apparent hydrodynamic diameters, were measured by DLS (Zetasizer Nano ZS90, Malvern Instruments Ltd., Worcestershire, Grovewood, UK). Suspensions of selenium nanoparticles were obtained as stated above and diluted 1:10 to meet the optical requirements of the instrument.

### Purity determination of selenium nanoparticles

A Varian SpectAA 220 FS (Mulgrave, Victoria, Australia) with a nitrous oxide-acetylene flame was use to determine the selenium purity (λ = 196,0 nm). A Merck Tritrisol Se standard (2000 ± 5) mg/L was use to prepare a calibration curve (0,00–100,0) mg/L. The selenium material was pretreated with HCl 3 M and then dissolved in supra pure HNO_3_ using an ultrasonic bath.

## Additional Information

**How to cite this article**: Avendaño, R. *et al*. Production of selenium nanoparticles in *Pseudomonas putida* KT2440. *Sci. Rep.* **6**, 37155; doi: 10.1038/srep37155 (2016).

**Publisher’s note:** Springer Nature remains neutral with regard to jurisdictional claims in published maps and institutional affiliations.

## References

[b1] Zhang W. . Biosynthesis and structural characteristics of selenium nanoparticles by *Pseudomonas alcaliphila*. Colloids Surf. B. Biointerfaces 88, 196–201 (2011). 21752611 10.1016/j.colsurfb.2011.06.031

[b2] Gates B., Mayers B., Cattle B. & Xia Y. Synthesis and characterization of uniform nanowires of trigonal selenium. Adv. Funct. Mater. 12, 219–227 (2002).

[b3] Stolz J. F. & Oremland R. S. Bacterial respiration of arsenic and selenium. FEMS Microbiol. Rev. 23, 615–627 (1999). 10525169 10.1111/j.1574-6976.1999.tb00416.x

[b4] Wadhwani S. A., Shedbalkar U. U., Singh R. & Chopade B. A. Biogenic selenium nanoparticles: current status and future prospects. Appl. Microbiol. Biotechnol. 100, 2555–2566 (2016). 26801915 10.1007/s00253-016-7300-7

[b5] Cai S. J. . Effects of nano-Selenium on performance, meat quality, immune function, oxidation resistance, and tissue selenium content in broilers. Poultry Science. 91, 2532–2539 (2012). 10.3382/ps.2012-02160 22991539

[b6] Shakibaie M., Forootanfar H., Golkari Y., Mohammadi-Khorsand T. & Shakibaie M. R. Anti-biofilm activity of biogenic selenium nanoparticles and selenium dioxide against clinical isolates of *Staphylococcus aureus, Pseudomonas aeruginosa*, and *Proteus mirabilis*. J. Trace Elem. Med. Biol. 29, 235–241 (2015). 25175509 10.1016/j.jtemb.2014.07.020

[b7] Tran P. A. & Webster T. J. Selenium nanoparticles inhibit *Staphylococcus aureus* growth. Int. J. Nanomedicine. 6, 1553–1558 (2011). 21845045 10.2147/IJN.S21729 PMC3152473

[b8] Cremonini E. . Biogenic selenium nanoparticles: characterization, antimicrobial activity and effects on human dendritic cells and fibroblasts. Microb. Biotechnol. 10.1111/1751-7915.12374 (2016). PMC5072192 27319803

[b9] Torres S. K. . Biosynthesis of selenium nanoparticles by *Pantoea agglomerans* and their antioxidant activity. J. Nanoparticle Res. 14, 1236 (2012).

[b10] Tapiero H., Townsend D. & Tew K. The antioxidant role of selenium and seleno-compounds. Biomed. Pharmacother. 57, 134–144 (2003). 12818475 10.1016/s0753-3322(03)00035-0 PMC6361120

[b11] Ike M., Takahashi K., Fujita T., Kashiwa M. & Fujita M. Selenate reduction by bacteria isolated from aquatic environment free from selenium contamination. Water Res. 34, 3019–3025 (2000).

[b12] Fishbein L. Environmental selenium and its significance. Toxicol. Sci. 3, 411–419 (1983). 10.1016/s0272-0590(83)80014-1 6357926

[b13] Lortie L., Gould W. D., Rajan S., McCready R. G. L. & Cheng K. J. Reduction of selenate and selenite to elemental selenium by a *Pseudomonas stutzeri* isolate. Appl. Environ. Microbiol. 58, 4042–4044 (1992). 16348829 10.1128/aem.58.12.4042-4044.1992 PMC183223

[b14] Oremland R. S. . Simultaneous reduction of nitrate and selenate by cell suspensions of selenium-respiring bacteria. Appl. Environ. Microbiol. 65, 4385–4392 (1999). 10508064 10.1128/aem.65.10.4385-4392.1999 PMC91582

[b15] Stolz J. F., Basu P. & Oremland R. S. Microbial transformation of elements: the case of arsenic and selenium. Int. Microbiol. 5, 201–207 (2002). 12497186 10.1007/s10123-002-0091-y

[b16] Steinberg N. A. & Oremland R. S. Dissimilatory selenate reduction potentials in a diversity of sediment types. Appl. Environ. Microbiol. 56, 3550–3557 (1990). 16348359 10.1128/aem.56.11.3550-3557.1990 PMC185022

[b17] Jora A. J. & Rastogi L. Biomimetric synthesis of selenium nanoparticles by *Pseudomonas aeruginosa* ATCC27853: An approach for conversion of selenite. J. Environ. Manage. 181, 231–236 (2016). 27353373 10.1016/j.jenvman.2016.06.029

[b18] Bajaj M., Schmidt S. & Winter J. Formation of Se(0) Nanoparticles by *Duganella sp*. and *Agrobacterium sp*. isolated from Se-laden soil of North-East Punjab, India. Microb. Cell Fact. 11, 1–14 (2012). 22607265 10.1186/1475-2859-11-64 PMC3391978

[b19] Yee N., Ma J., Dalia A., Boonfueng T. & Kobayashi D. Y. Se(VI) reduction and the precipitation of Se(0) by the facultative bacterium *Enterobacter cloacae* SLD1a-1 are regulated by FNR. Appl. Environ. Microbiol. 73, 1914–1920 (2007). 17261520 10.1128/AEM.02542-06 PMC1828800

[b20] Switzer J. . *Bacillus arsenicoselenatis*, sp. nov., and *Bacillus selenitireducens*, sp. nov.: Two haloalkaliphiles from Mono Lake, California that respire oxyanions of selenium and arsenic. Arch. Microbiol. 171, 19–30 (1998). 9871015 10.1007/s002030050673

[b21] Tugarova A. V. . Reduction of selenite by *Azospirillum brasilense* with the formation of selenium nanoparticles. Microb. Ecol. 68, 495–503 (2014). 24863127 10.1007/s00248-014-0429-y

[b22] Debieux C. M. . A bacterial process for selenium nanosphere assembly. Proc. Natl. Acad. Sci. USA 108, 13480–13485 (2011). 21808043 10.1073/pnas.1105959108 PMC3158160

[b23] Fernández-Llamosas H., Castro L., Blázquez M. L., Díaz E. & Carmona M. Biosynthesis of selenium nanoparticles by *Azoarcus sp.* CIB. Microb. Cell Fact. 15, 109 (2016). 27301452 10.1186/s12934-016-0510-y PMC4908764

[b24] Sabaty M., Avazeri C., Pignol D. & Vermeglio A. Characterization of the reduction of selenate and tellurite by nitrate reductases. Appl. Environ. Microbiol. 67, 5122–5126 (2001). 11679335 10.1128/AEM.67.11.5122-5126.2001 PMC93280

[b25] Schröder I., Rech S., Krafft T. & Macy J. M. Purification and characterization of the selenate reductase from *Thauera selenatis*. J. Biol. Chem. 272, 23765–23768 (1997). 9295321 10.1074/jbc.272.38.23765

[b26] Macy J. M. . *Thauera selenatis* gen. nov., sp. nov., a member of the beta subclass of Proteobacteria with a novel type of anaerobic respiration. Int. J. Syst. Bacteriol. 43, 135–142 (1993). 8427805 10.1099/00207713-43-1-135

[b27] Butler C. S., Debieux C. M., Dridge E. J., Splatt P. & Wright M. Biomineralization of selenium by the selenate-respiring bacterium *Thauera selenatis*. Biochem. Soc. Trans. 40, 1239–1243 (2012). 23176461 10.1042/BST20120087

[b28] Kessi J. & Hanselmann K. W. Similarities between the abiotic reduction of selenite with glutathione and the dissimilatory reaction mediated by *Rhodospirillum rubrum* and *Escherichia coli*. J. Biol. Chem. 279, 50662–50669 (2004). 15371444 10.1074/jbc.M405887200

[b29] Bébien M. . Involvement of superoxide dismutases in the response of *Escherichia coli* to selenium oxides. J. Bacteriol. 184, 1556–1564 (2002). 11872706 10.1128/JB.184.6.1556-1564.2002 PMC134873

[b30] Bébien M., Chauvin J., Adriano J., Grosse S. & Verméglio A. Effect of selenite on growth and protein synthesis in the phototrophic bacterium *Rhodobacter sphaeroides*. Appl. Environ. Microbiol. 67, 4440–4447 (2001). 11571140 10.1128/AEM.67.10.4440-4447.2001 PMC93187

[b31] Peng D., Zhang J., Liu Q. & Taylor E. W. Size effect of elemental selenium nanoparticles (Nano-Se) at supranutritional levels on selenium accumulation and glutathione S-transferase activity. J. Inorg. Biochem. 101, 1457–1463 (2007). 17664013 10.1016/j.jinorgbio.2007.06.021

[b32] Lampis S. . Delayed formation of zero-valent selenium nanoparticles by *Bacillus mycoides* SeITE01 as a consequence of selenite reduction under aerobic conditions. Microb. Cell Fact. 13, 35 (2014). 24606965 10.1186/1475-2859-13-35 PMC3975340

[b33] Prakash N. T. . Aerobic microbial manufacture of nanoscale selenium: exploiting nature’s bionanomineralization potential. Biotechnol. Lett. 31, 1857 (2009). 19690806 10.1007/s10529-009-0096-0

[b34] Pearce C. I. . Microbial manufacture of chalcogenide-based nanoparticles via the reduction of selenite using *Veillonella atypica*: an *in situ* EXAFS study. Nanotechnology. 19, 155603 (2008). 21825617 10.1088/0957-4484/19/15/155603

[b35] Nelson K. E. . Complete genome sequence and comparative analysis of the metabolically versatile *Pseudomonas putida* KT2440. Environ. Microbiol. 4, 799–808 (2002). 12534463 10.1046/j.1462-2920.2002.00366.x

[b36] Plaggenborg R., Overhage J., Steinbüchel A. & Priefert H. Functional analyses of genes involved in the metabolism of ferulic acid in *Pseudomonas putida* KT2440. Appl. Microbiol. Biotechnol. 61, 528–535 (2003). 12764569 10.1007/s00253-003-1260-4

[b37] Chavarría M., Nikel P. I., Pérez-Pantoja D. & De Lorenzo V. The Entner-Doudoroff pathway empowers *Pseudomonas putida* KT2440 with a high tolerance to oxidative stress. Environ. Microbiol. 15, 1772–1785 (2013). 23301697 10.1111/1462-2920.12069

[b38] Nikel P. I., Chavarria M., Fuhrer T., Sauer U. & de Lorenzo V. *Pseudomonas putida* KT2440 metabolizes glucose through a cycle formed by enzymes of the Entner-Doudoroff, Embden-Meyerhof-Parnas, and pentose phosphate pathways. J. Biol. Chem. 290, 25920–25932 (2015). 26350459 10.1074/jbc.M115.687749 PMC4646247

[b39] Nikel P. I., Chavarría M., Danchin A. & de Lorenzo V. From dirt to industrial applications: *Pseudomonas putida* as a Synthetic Biology chassis for hosting harsh biochemical reactions. Curr. Opin. Chem. Biol. 34, 20–29 (2016). 27239751 10.1016/j.cbpa.2016.05.011

[b40] Rech S. A. & Macy J. M. The terminal reductases for selenate and nitrate respiration in *Thauera selenatis* are two distinct enzymes. J. Bacteriol. 174, 7316–7320 (1992). 1429454 10.1128/jb.174.22.7316-7320.1992 PMC207426

[b41] Isalan M. . Evolvability and hierarchy in rewired bacterial gene networks. Nature. 452, 840–845 (2008). 18421347 10.1038/nature06847 PMC2666274

[b42] Silva-Rocha R., Chavarría M., Kleijn R. J., Sauer U. & de Lorenzo V. The IHF regulon of exponentially growing *Pseudomonas putida* cells. Environ. Microbiol. 15, 49–63 (2013). 22510163 10.1111/j.1462-2920.2012.02750.x

[b43] Dhanjal S. & Cameotra S. S. Aerobic biogenesis of selenium nanospheres by *Bacillus cereus* isolated from coalmine soil. Microb. Cell Fact. 9, 52 (2010). 20602763 10.1186/1475-2859-9-52 PMC2909957

[b44] Zheng S. . Selenite reduction by the obligate aerobic bacterium *Comamonas testosteroni* S44 isolated from a metal-contaminated soil. BMC Microbiol. 14, 204 (2014). 25098921 10.1186/s12866-014-0204-8 PMC4236595

[b45] Cantafio A. W. . Pilot-scale selenium bioremediation of San Joaquin drainage water with *Thauera selenatis*. Appl. Environ. Microbiol. 62, 3298–3303 (1996). 16535401 10.1128/aem.62.9.3298-3303.1996 PMC1388939

[b46] Sambrook J. & Russell D. W. Molecular cloning: a laboratory manual. (eds. Sambrook J. & Russell D. W.) A2.2 (Cold Spring Harbor, 2001).

[b47] Miller J. H. Experiments in molecular genetics. (ed. Miller J. H.) 431 (Cold Spring Harbor, 1974).

[b48] Karnovsky M. J. A formaldehyde-glutaraldehyde fixative of high osmolarity for use in electron microscopy. J. Cell Biol. 27, 137A (1965).

